# P90RSK and Nrf2 Activation via MEK1/2-ERK1/2 Pathways Mediated by Notoginsenoside R2 to Prevent 6-Hydroxydopamine-Induced Apoptotic Death in SH-SY5Y Cells

**DOI:** 10.1155/2013/971712

**Published:** 2013-09-18

**Authors:** Xiang-Bao Meng, Gui-Bo Sun, Min Wang, Jing Sun, Meng Qin, Xiao-Bo Sun

**Affiliations:** Key Laboratory of Bioactive Substances and Resources Utilization of Chinese Herbal Medicine, Ministry of Education, Institute of Medicinal Plant Development, Chinese Academy of Medical Sciences and Peking Union Medical College, No. 151, Malianwa North Road, Haidian District, Beijing 100193, China

## Abstract

6-Hydroxydopamine (6-OHDA) is known to contribute to neuronal death in Parkinson's disease. In this study, we found that the preincubation of SH-SY5Y cells for 24 h with 20 **μ**M notoginsenoside R2 (NGR2), which is a newly isolated notoginsenoside from *Panax notoginseng*, showed neuroprotective effects against 6-OHDA-induced oxidative stress and apoptosis. NGR2 incubation successively resulted in the activation of P90RSK, inactivation of BAD, and inhibition of 6-OHDA-induced mitochondrial membrane depolarization, thus preventing the mitochondrial apoptosis pathway. NGR2 incubation also led to the activation of Nrf2 and subsequent activity enhancement of phase II detoxifying enzymes, thus suppressing 6-OHDA-induced oxidative stress, and these effects could be removed by Nrf2 siRNA. We also found that the upstream activators of P90RSK and Nrf2 were the MEK1/2–ERK1/2 pathways but not the JNK, P38, or PI3K/Akt pathways. Interestingly, NGR2 incubation could also activate MEK1/2 and ERK1/2. Most importantly, NGR2-mediated P90RSK and Nrf2 activation, respective downstream target activation, and neuroprotection were reversed by the genetic silencing of MEK1/2 and ERK1/2 by using siRNA and PD98059 application. These results suggested that the neuroprotection elicited by NGR2 against 6-OHDA-induced neurotoxicity was associated with NGR2-mediated P90RSK and Nrf2 activation through MEK1/2-ERK1/2 pathways.

## 1. Introduction

Parkinson's disease (PD) is a neurodegenerative disorder characterized by severe motor deficits including resting tremor, rigidity, bradykinesia, and postural instability. The pathophysiological changes responsible for these motor deficits are known to be associated with the selective loss of dopaminergic neurons in the substantia nigra pars compacta and subsequent depletion of striatal dopamine content.

To date, although L-dopa or MAO-B inhibitors such as rasagiline show symptomatic relief, no available therapy can delay or halt the neurodegenerative process of PD [1]. Therefore, an urgent clinical need exists for effective PD drugs and therapies. A promising effective therapy for PD may be achieved by target- and mechanism-based drug development [2]. Previous studies consistently implicated that oxidative stress and mitochondrial dysfunction are common mechanisms that lead to the demise of dopaminergic neurons in both familial and sporadic PD [3, 4]. Neuroprotective therapies are presumed approaches to suppress oxidative stress and reverse mitochondrial dysfunction in PD.

We mimicked the pathogenesis of PD by using an *in vitro* PD model of 6-OHDA-induced cell death in SH-SY5Y cells [5]. SH-SY5Y cells are widely used for studies of neuronal survival, apoptosis, and their underlying mechanisms [6], and 6-OHDA can lead to oxidative stress, mitochondrial dysfunction, and apoptosis in SH-SY5Y cells [7]. This model can be used to determine the possible neuroprotective compounds and investigate the underlying mechanism.

**Figure 1:**
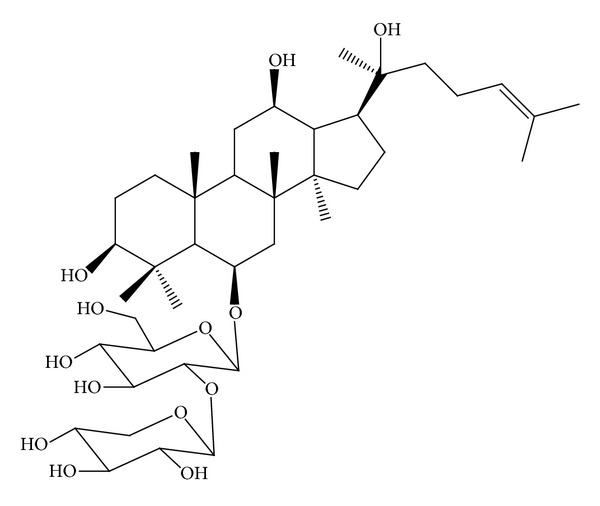
Chemical structure of NGR2.

*P. notoginseng* saponins, the main active ingredients of *P. notoginseng* (Burk.) F. H. Chen, have neuroprotective properties against PD *in vivo* [8]. Furthermore, ginsenosides Rg1 [9] and Re [10], which are the main saponins in *P. notoginseng* saponins, protect dopaminergic neurons *in vivo*. Notoginsenoside R2 (NGR2), whose chemical structure is shown in Figure 1, is a new notoginsenoside isolated from *P. notoginseng*. We hypothesized that NGR2 might be neuroprotective because NGR2 has a similar chemical structure to ginsenosides Rg1 and Re. However, the neuroprotective properties of NGR2 are largely unknown. Since our preliminary experiments found that NGR2 has an ability to protect neurons against various toxic stimuli, in the present study, multiple approaches were conducted to explore the neuroprotective effects of NGR2 and underlying mechanisms.

Phase II detoxifying enzymes including heme oxygenase-1 (HO-1), glutathione peroxidase (GSH-PX), and glutathione peroxidase (GR) are believed to play a central role in neuronal defense [11]. They can reportedly be upregulated in neurons via the activation of nuclear factor-erythroid 2-related factor 2 (Nrf2) by pharmacological inducers such as ginsenoside Rg1 [12]. Moreover, activated P90RSK can phosphorylate and inactivate a proapoptotic protein BAD [13] and subsequently inhibit the apoptotic pathways, which emphasizes the importance of P90RSK. Targeting the P90RSK and Nrf2 signaling pathways may provide neuroprotection advantages. Furthermore, P90RSK and Nrf2 activation is regulated by multiple signaling pathways such as ERK1/2, JNK, p38, and PI3K/Akt [14–20]. Whether these mechanisms are responsible for NGR2-mediated neuroprotection remains to be seen.

In this study, we demonstrated that NGR2 possessed neuroprotective effects against 6-OHDA-induced apoptotic death in SH-SY5Y cells by the activation of P90RSK and Nrf2 via MEK1/2-ERK1/2 signaling pathways.

## 2. Materials and Methods

### 2.1. Materials

NGR2 (molecular weight = 871.01; purity > 98%) was purchased from Shanghai Winherb Medical S&T Development (China). Human SH-SY5Y neuroblastoma cell line was obtained from the Cell Resource Center of the Institute of Basic Medical Sciences, Peking Union Medical College/Chinese Academy of Medical Sciences (China). Dulbecco's modified Eagle's medium (DMEM) and fetal bovine serum (FBS) were purchased from Gibco Life Technologies (USA). Primary and secondary antibodies were all purchased from Santa Cruz Biotechnology (USA). All other chemicals used were obtained from Sigma (USA).

### 2.2. Cell Culture

SH-SY5Y cells were maintained in a 1 : 1 mixture of F12 nutrient and DMEM supplemented with 10% (v/v) heat-inactivated FBS, 2 mM L-glutamine, 50 U/mL penicillin, and 50 *μ*g/mL streptomycin. Rat pheochromocytoma PC12 cells were grown in DMEM that contains 10% heat-inactivated horse serum, 5% heat-inactivated FBS, 50 U/mL penicillin, and 50 *μ*g/mL streptomycin. SH-SY5Y and PC12 cells were maintained at 37°C in a humidified atmosphere of 95% air and 5% CO_2_. Cells at a passage below 10 and in the exponential growth phase were used in all experiments. Primary cortical neurons were prepared from embryonic day 18 Sprague-Dawley rat fetuses according to a previously described method with a slight modification [21]. The protocol was performed according to the Guide for the Care and Use of Laboratory Animals of the National Institute of Health and approved by the Animal Ethics Committee of Peking Union Medical College. All efforts were made to minimize the number of animals used and reduce their suffering. The cerebral cortex was collected, mechanically fragmented, and incubated at 37°C for 10 min with 0.125% trypsin. The cortical neurons were incubated in DMEM/F12 supplemented with 20% heat-inactivated FBS for 4 h in a humidified atmosphere of 95% air and 5% CO_2_ at 37°C. After the cells attached to the substrate, the culture medium was replaced with a serum-free neurobasal medium supplemented with 2% B27 (Gibco, USA) and changed twice a week. The experiments were performed on day seven of the cell culture.

### 2.3. Drug Preparation

NGR2 was stored at 4°C as a stock solution (100 mM) in dimethyl sulfoxide (DMSO). 6-OHDA was dissolved in sterile distilled water containing 0.1% ascorbic acid as a stock solution (1 M). NGR2 and 6-OHDA stock solutions were diluted in DMEM/F-12 immediately before use.

### 2.4. Analysis of Cell Viability and Morphological Changes

Cell viability was determined by cell counting kit-8 (Dojindo Laboratories, Japan). SH-SY5Y cells were cultured in 96-well plates at a density of 1 × 10^4^ cells/well and grown for 24 h. The cells were treated with 6-OHDA or preincubated with NGR2 followed by treatment with 6-OHDA or coincubation with NGR2 and 6-OHDA. Cells incubated in DMEM/F12 that contain an equivalent concentration of DMSO (the highest concentration less than 0.1%) were used as the control. Thereafter, a 10 *μ*L cell counting kit-8 solution was added to each well. The absorbance was detected at 450 nm on a microplate reader after 1 h (Spectra Fluor, Tecan, Sunrise, Austria). Cell viability was expressed as a percentage of the control. The morphological changes in the SH-SY5Y cells were visualized by an inverted microscope connected to a digital camera (Canon, Japan).

### 2.5. Quantization of Cell Apoptosis Rate

Cell apoptosis was determined by flow cytometry by using Annexin V-propidium iodide (PI) double staining kits (Invitrogen, USA). SH-SY5Y cells (5 × 10^4^ cells/well) were cultured in 24-well plates. The cells were preincubated with 20 *μ*M NGR2 for 24 h followed by treatment with 50 *μ*M 6-OHDA for 24 h. Then, the cells were washed twice with ice-cold PBS and collected by trypsinization and centrifugation. The cells were incubated in the dark in 100 *μ*L of 1× binding buffer with 5 *μ*L Annexin V and 1 *μ*L PI for 15 min. After 400 *μ*L of 1× binding buffer was added, the cells were subjected to FACSCalibur analysis (BD Biosciences, USA).

### 2.6. Detection of Cell DNA Fragmentation

The DNA fragmentation in the apoptotic SH-SY5Y cells was detected by TUNEL assay by using ApopTag Fluoresce *in situ* Apoptosis Detection Kits (Millipore, MA, USA). SH-SY5Y cells were cultured on cover slips. The cells were preincubated with 20 *μ*M NGR2 for 24 h followed by treatment with 50 *μ*M 6-OHDA for 24 h. The cells were washed twice with PBS and fixed in 4% neutral-buffered formalin solution for 30 min. The cells were rinsed with PBS and incubated with a methanol solution containing 0.3% H_2_O_2_ for 30 min. Thereafter, the cells were incubated by a permeabilizing solution (0.1% sodium citrate and 0.1% Triton X-50) for 10 min. After rinsing in the equilibration buffer, the cells were incubated with a working-strength TdT enzyme in a humidified chamber at 37°C for 1 h. The cells were then rinsed in the stop/wash buffer and incubated with the working-strength antidigoxigenin conjugate at room temperature for 30 min. After washing in PBS, the cells were counterstained by diamidino-2-phenylindole (DAPI) and viewed under a fluorescence microscope (Leica, Germany).

### 2.7. Detection of Intracellular ROS

To detect intracellular reactive oxygen species (ROS), the molecular probe 5-(and -6)-carboxy-2′,7′-dichlorodihydrofluorescein diacetate (carboxy-H2DCFDA) was used. SH-SY5Y cells (1 × 10^4^ cells/well) were cultured in 96-well plates. The cells were preincubated with 20 *μ*M NGR2 for 24 h followed by treatment with 50 *μ*M 6-OHDA for 24 h. The cells were harvested and washed with 1× washing buffer and incubated with carboxy-H2DCFDA (final concentration of 25 *μ*M) in the dark at 37°C for 30 min. The fluorescence was immediately detected on a microplate reader. Excitation and emission wavelengths were 495 and 529 nm, respectively. The level of cellular ROS was expressed as a percent of the control.

### 2.8. Determination of LDH and MDA Levels and HO-1, GSH-PX, and GR Activities

Lactate dehydrogenase (LDH) and malondialdehyde (MDA) levels, as well as GSH-PX and GR activities, were measured by using the respective assay kits according to the manufacturer's protocol (Nanjing Jiancheng Bioengineering Institute, China). HO-1 activities were determined by using HO-1 ELISA kits (RapidBio Lab, USA). SH-SY5Y cells (1 × 10^5^ cells/well) were cultured in 6-well plates. The cells were preincubated with 20 *μ*M NGR2 for 24 h followed by treatment with 50 *μ*M 6-OHDA for 24 h. The cell culture media were collected to measure the level of extracellular LDH. The cells were harvested to measure the level of intracellular LDH and MDA and detect HO-1, GSH-PX, and GR activities. LDH release was expressed as the rate of extracellular LDH to total LDH (intracellular plus extracellular). HO-1 activity was expressed as the fold change of the control.

### 2.9. Measurement of Mitochondrial Membrane Potential

JC-1 (5′,6,6′-tetrachloro-1,1′,3,3′-tetraethylbenzimidazolylcarbocyanine iodide, Enzo Life Sciences International, USA) was used to measure the changes in mitochondrial membrane potential. SH-SY5Y cells (1 × 10^5^ cells/well) were cultured in 6-well plates. The cells were preincubated with 20 *μ*M NGR2 for 24 h followed by treatment with 50 *μ*M 6-OHDA for 24 h. The cells were washed with PBS and incubated in the dark at 37°C with JC-1 (final concentration of 2 *μ*M) for 30 min. After washing twice with PBS, the cells labeled with JC-1 were analyzed by a high content screening system (Molecular devices, USA).

### 2.10. siRNA Transfection

MEK1/2 siRNA, ERK1/2 siRNA, Nrf2 siRNA, and control siRNA were purchased from Santa Cruz Biotechnology (USA). SH-SY5Y cells were cultured in 6-well plates. When 50% confluence was achieved, SH-SY5Y cells were transfected with MEK1/2 siRNA (100 nM), ERK1/2 siRNA (100 nM), Nrf2 siRNA (50 nM), or equivalent concentrations of control siRNA by using a Lipofectamine 2000 reagent (Invitrogen, USA). The cells were transfected for 48 h in Opi-MEM medium without serum and antibiotics and incubated with 20 *μ*M NGR2 for 24 h.

### 2.11. Preparation of Cytosolic and Nuclear Proteins

Reagents and kits used were all purchased from Santa Cruz Biotechnology (USA). Cytosolic and nuclear proteins were prepared by using cell nuclear protein extraction kits. SH-SY5Y cells were briefly washed with cold PBS, scraped in cold buffer A supplemented with protease inhibitor cocktail, and incubated on ice for 15 min. The supernatant that contains cytosolic proteins was collected by centrifugation at 12,000 rpm for 10 min at 4°C. A pellet-containing nuclear fraction was re-suspended in buffer *C* supplemented with a protease inhibitor cocktail and incubated on ice for 30 min. Thereafter, the supernatant that contains nuclear proteins was collected by centrifugation at 12,000 rpm for 10 min at 4°C. The total protein concentration was determined by bicinchoninic acid assay kits and boiled with a sodium dodecyl sulfate-polyacrylamide gel electrophoresis loading buffer for 5 min. The cytosolic and nuclear proteins were stored at −80°C until use.

### 2.12. Western Blot Analysis

Protein expression was determined by western blot analysis. Briefly, equal amounts of protein were separated by electrophoresis on 10% SDS polyacrylamide gels and transferred onto nitrocellulose membranes. The membranes were incubated with 5% (w/v) nonfat milk powder in tris-buffered saline containing 0.1% (v/v) Tween 20 for 2 h to block nonspecific binding sites. Thereafter, the membranes were incubated overnight at 4°C with the respective primary antibodies. The primary antibodies used were as follows: rabbit polyclonal anti-p-MEK-1/2 (Ser218/Ser222); rabbit polyclonal anti-MEK-1/2 (12-B); rabbit polyclonal anti-p-ERK1/2 (Thr177/Thr160)-R; mouse monoclonal anti-ERK 1/2 (MK1); rabbit polyclonal anti-p-Rsk-1 (Ser363); mouse monoclonal anti-Rsk (B-4); rabbit polyclonal anti-Nrf2 (C-20); Lamin A (H-102): sc-20680; goat polyclonal anti-p-BAD (Ser112); rabbit polyclonal anti-BAD (C-20); mouse monoclonal anti-Bcl-XL (H-5); rabbit polyclonal anti-BAX (N-20); mouse monoclonal anticytochrome C (6H2); goat polyclonal anticleaved caspase-9 p10 (h331); goat polyclonal anticleaved caspase-3 p11 (h176); mouse monoclonal anti-*β*-actin (C4). After washing with tris-buffered saline and Tween 20 (TBST), the membranes were incubated for 1 h at room temperature with the respective peroxidase-conjugated secondary antibodies. The membranes were rewashed with TBST, and the bands were developed by using an enhanced chemiluminescence reagent. The protein levels were quantified by densitometry by using Image J software.

### 2.13. Statistical Analysis

The data were expressed as the mean ± standard deviation (SD) of three independent experiments. ANOVA followed by the Newman-Keuls post hoc test or Student's *t*-test were used for multiple group comparison and two-group comparison, respectively. *P* < 0.05 was considered statistically significant.

## 3. Results

### 3.1. NGR2 Inhibited 6-OHDA-Induced Cell Death in SH-SY5Y Cells

The potential protective effects of NGR2 were investigated in SH-SY5Y cells exposed to 6-OHDA.

**Figure 2:**
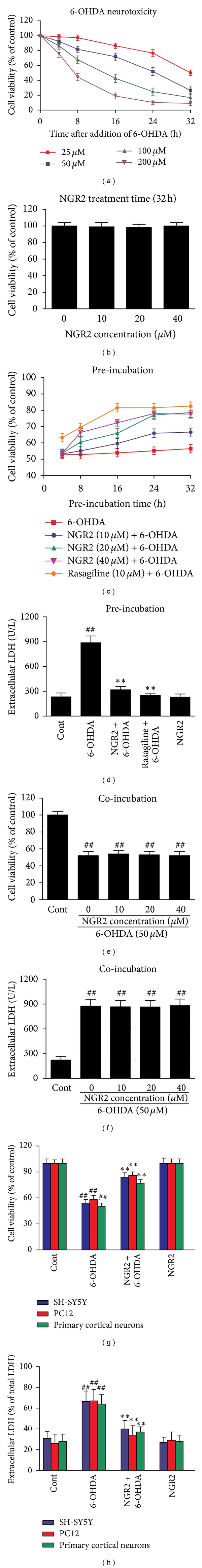
Protective effects of NGR2 on 6-OHDA-induced cell death in SH-SY5Y cells. Cell viability was measured by cell counting kit-8 test or LDH assay. (a) 6-OHDA could induce cell death in SH-SY5Y cells in concentration- and time-dependent manners. (b) NGR2 had no toxic effect on cell viability. Preincubation with different concentrations of NGR2 for different periods of time had protective effect on 6-OHDA-induced cell death (c) and LDH release (d) in SH-SY5Y cells. Coincubation with NGR2 had almost no protective effect on 6-OHDA-induced cell death (e) and LDH release (f) in SH-SY5Y cells. Both the cell counting kit-8 test (g) and LDH assay (h) showed that the neuroprotective effects of NGR2 was independent of cell type. The results were expressed as the mean ± SD of three independent experiments. ## indicates a significant difference from the control (*P* < 0.01). ∗∗ indicates a significant difference from the 6-OHDA treatment alone (*P* < 0.01).

First, we investigated the effect of 6-OHDA on SH-SY5Y cells. The SH-SY5Y cell treatment at different concentrations (25, 50, 100, and 200 *μ*M) of 6-OHDA for various periods (4, 8, 16, 24, and 32 h) decreased cell viability in concentration- and time-dependent manners (Figure 2(a)). Cell viability was reduced approximately to 50% of the control when SH-SY5Y cells were exposed to 50 *μ*M of 6-OHDA for 24 h. Thus, the concentration (50 *μ*M) and period (24 h) were used for further investigations.

Second, we investigated the effect of NGR2 on SH-SY5Y cells. The results indicated that no significant difference in cell viability was found when SH-SY5Y cells were incubated for 32 h at different concentrations (10, 20, and 40 *μ*M) of NGR2 compared with the control. This finding suggested that NGR2 had no toxic effect on SH-SY5Y cells (Figure 2(b)).

Third, we explored the potential protective effect of NGR2 on 6-OHDA-induced cell death in SH-SY5Y cells. The pre-incubation of SH-SY5Y cells with increasing concentrations (10, 20, and 40 *μ*M) of NGR2 for different time periods (4, 8, 16, and 24 h) reversed the decreased cell viability induced by 6-OHDA (Figure 2(c)). In this study, rasagiline was used as a positive control drug for it is a specific monoamine oxidase B inhibitor and used as a monotherapy in early Parkinson's disease or as an adjunct therapy in more advanced cases. As shown in Figure 2(c), the pre-incubation of SH-SY5Y cells with 10 *μ*M of rasagiline for different time periods (4, 8, 16, and 24 h) markedly inhibited the reduction of cell viability induced by 6-OHDA. However, NGR2 pre-incubation of SH-SY5Y cells with 40 *μ*M NGR2 for 32 h had almost no incremental protective action compared with that of 20 *μ*M NGR2 for 24 h, and the cell viability resumed to 80% of control. This result suggested that the maximum protective effect was achieved with 20 *μ*M NGR2 for 24 h. Nevertheless, the maximum protective effect of NGR2 is lower than rasagiline. Therefore, the concentration (20 *μ*M) and time period (24 h) were selected for further investigations.

We confirmed the protective effect of NGR2 by using LDH release assay. SH-SY5Y cell pre-incubation with 20 *μ*M of NGR2 or 10 *μ*M of rasagiline for 24 h decreased LDH release in cells treated with 6-OHDA (Figure 2(d)). In contrast, NGR2 treatment alone had no effect on LDH release.

Both the cell counting kit-8 test (Figure 2(e)) and LDH assay (Figure 2(f)) demonstrated that almost no protection was obtained when NGR2 (10, 20, and 40 *μ*M) was co-incubated with 6-OHDA for 24 h. This finding suggested that the protective effect of NGR2 was exerted only by pretreatment and not by co-treatment with 6-OHDA.

Finally, we assessed whether the observed neuroprotective action of NGR2 was cell type specific by using pheochromocytoma PC12 cells and rat primary cortical neurons. The neuroprotection of NGR2 against 6-OHDA toxic effects was confirmed in these two types of cells by both cell counting kit-8 test (Figure 2(g)) and LDH release assay (Figure 2(h)). This result suggested that the neuroprotective effect of NGR2 was independent of cell type.

### 3.2. NGR2 Ameliorated 6-OHDA-Induced Morphological Changes in SH-SY5Y Cells

**Figure 3:**
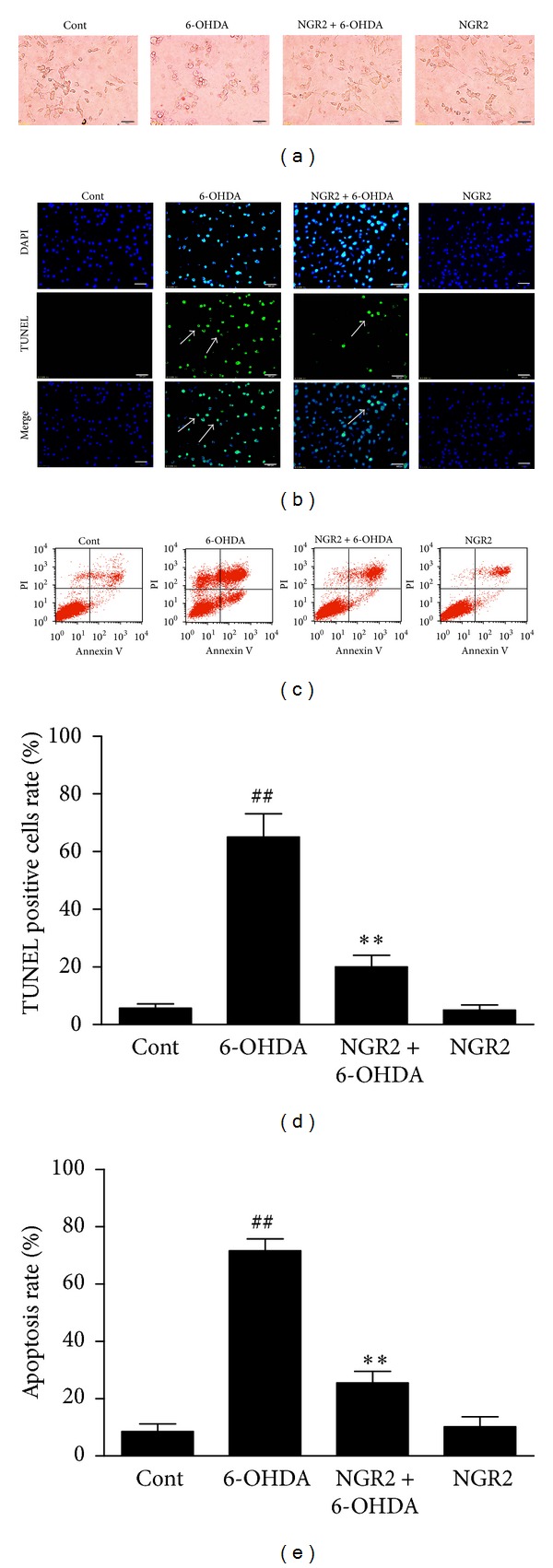
Protective effect of NGR2 on 6-OHDA-induced apoptosis in SH-SY5Y cells. The SH-SY5Y cells were preincubated with 20 *μ*M NGR2 for 24 h followed by treatment with 50 *μ*M 6-OHDA for 24 h. (a) Photographs of morphological changes in SH-SY5Y cells were visualized by an inverted microscope connected to a digital camera, bar = 50 *μ*m. (b) Photographs of DNA fragmentation were detected by TUNEL assay in the apoptotic SH-SY5Y cells, bar = 50 *μ*m. Arrows represent TUNEL-positive cells. (c) Cell apoptosis was determined by Annexin V-PI double staining kits by using flow cytometry. (d) Quantification of the TUNEL-positive cell rate. (e) Quantification of the apoptosis rate. The results were expressed as the mean ± SD of three independent experiments. ## indicates a significant difference from the control (*P* < 0.01). ∗∗ indicates a significant difference from the 6-OHDA treatment alone (*P* < 0.01).

The control SH-SY5Y cells had normal shapes with a smooth cellular profile and extensive neurite processes, whereas cells treated with 6-OHDA lost their neurite processes and became round (Figure 3(a)). An obvious amelioration of the morphological changes induced by 6-OHDA was observed in cells preincubated with NGR2. However, NGR2 treatment alone did not alter the morphology of the SH-SY5Y cells.

### 3.3. NGR2 Inhibited 6-OHDA-Induced Apoptosis in SH-SY5Y Cells

DNA fragmentation is a typical marker of apoptosis. Therefore, the nuclear fragmentation in apoptotic cells was detected to investigate the possible effects of NGR2 on 6-OHDA-induced apoptosis. The DNA fragmentation and TUNEL-positive cell rate were dramatically augmented in SH-SY5Y cells exposed to 6-OHDA compared with the control (Figures 3(b) and 3(d)). In contrast, pretreatment with NGR2 effectively reversed these changes induced by 6-OHDA, but NGR2 treatment alone had no effect on DNA fragmentation. We then corroborated the protective effect of NGR2 in SH-SY5Y cells by Annexin V-PI double staining by using flow cytometry. An early indicator of apoptosis is the translocation of the membrane phospholipid phosphatidylserine from the cytoplasmic interface to the extracellular surface. The phospholipid phosphatidylserine that accumulates on the extracellular surface can be detected by Annexin V. PI is a fluorescent dye that binds to the nuclei of dead cells. Annexin−/PI−, Annexin+/PI−, and Annexin+/PI+ represented the viable cells, early apoptotic cells, and late apoptotic cells, respectively. Figures 3(c) and 3(e) show that Annexin+/PI− and Annexin+/PI+ substantially increased in the 6-OHDA-treated cells. This result suggested that the apoptosis rate was significantly increased when SH-SY5Y cells were challenged by 6-OHDA. However, these changes were markedly reversed by NGR2 pre-incubation. NGR2 treatment alone had no effect on the apoptosis rate. These results suggested that NGR2 was capable of rescuing SH-SY5Y cells from 6-OHDA-induced apoptotic death.

### 3.4. NGR2 Activated P90RSK and Nrf2 Pathways in SH-SY5Y Cells

**Figure 4:**
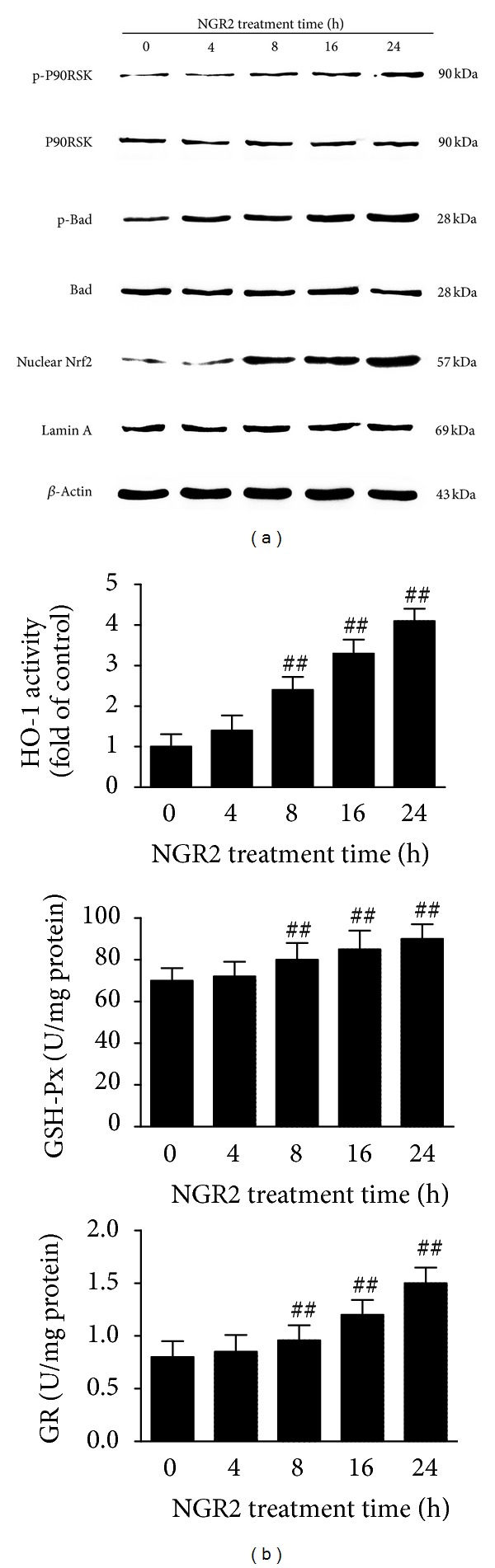
NGR2 could activate P90RSK and Nrf2 pathways in SH-SY5Y cells. The expression of proteins was determined by western blot analysis. HO-1 activities were measured by HO-1 ELISA kits. GSH-PX and GR activities were measured by GSH-PX and GR assay kits, respectively. The SH-SY5Y cells were treated with 20 *μ*M NGR2 for different periods of time (4, 8, 16, and 24 h). (a) NGR2 time dependently increased p-P90RSK and p-BAD expression and nuclear Nrf2 accumulation. (b) NGR2 increased the activities of HO-1, GSH-PX, and GR in a time-dependent manner. The results are expressed as the mean ± SD of three independent experiments. ## indicates a significant difference from the control (*P* < 0.01).

Given that P90RSK and Nrf2 activation has beneficial effects on cell survival, we examined the effect of NGR2 on P90RSK and Nrf2 activation in SH-SY5Y cells. The time-dependent stimulation of SH-SY5Y cells with NGR2 increased the phosphorylation of P90RSK and the subsequent phosphorylation of BAD, which is a downstream target of activated P90RSK. SH-SY5Y cells receiving NGR2 similarly exhibited enhanced levels of nuclear Nrf2 accumulation (Figure 4(a)) and subsequent increase in phase II detoxifying enzyme activities such as HO-1, GSH-PX, and GR in a time-dependent manner (Figure 4(b)).

### 3.5. NGR2 Inhibited 6-OHDA-Induced Oxidative Stress in SH-SY5Y Cells

**Figure 5:**
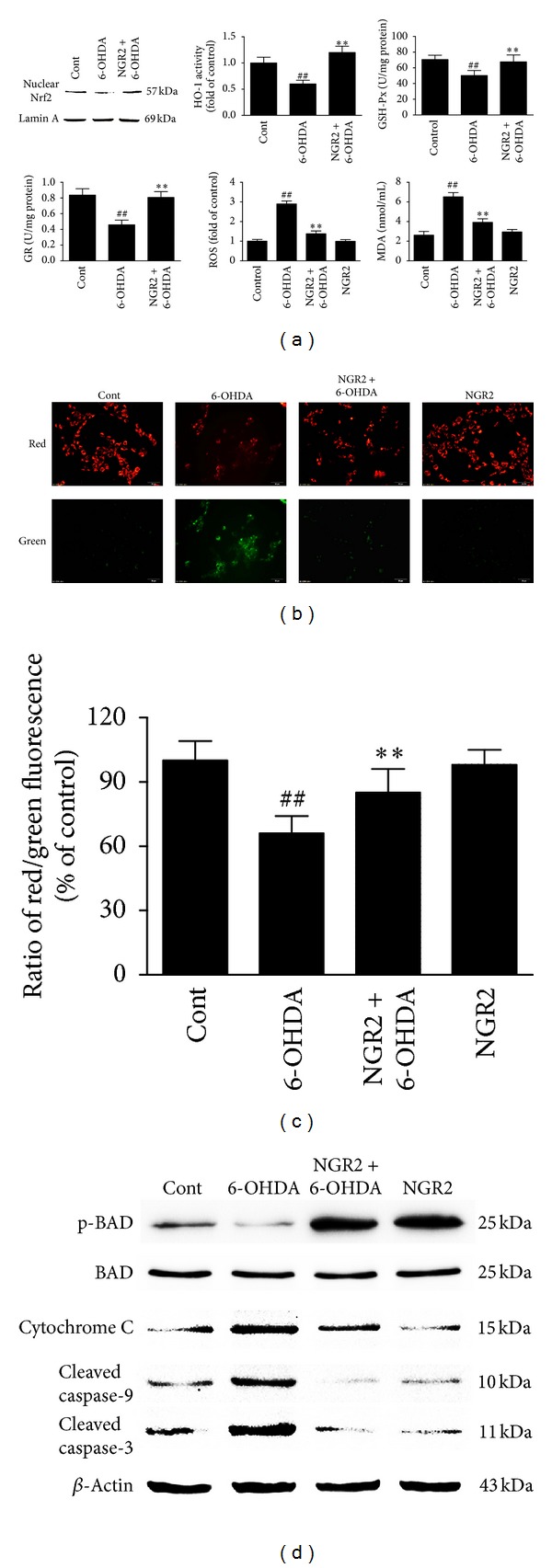
NGR2 reversal of 6-OHDA-induced oxidative stress, mitochondrial membrane depolarization, and apoptosis-related protein deregulation in SH-SY5Y cells. The SH-SY5Y cells were preincubated with 20 *μ*M NGR2 for 24 h followed by treatment with 50 *μ*M 6-OHDA for 24 h. (a) NGR2 reversal of 6-OHDA induced the increase in MDA production and ROS generation, as well as the decrease of nuclear Nrf2, HO-1, GSH-PX, and GR activities in SH-SY5Y cells. (b) NGR2 reversal of mitochondrial membrane depolarization. (c) The cells labeled by JC-1 were analyzed by a high content screening system. (d) NGR2 suppression of 6-OHDA induced increase in cytochrome c release, upregulation of cleaved caspase-9 and cleaved caspase-3, and downregulation of p-BAD. The results are expressed as the mean ± SD of three independent experiments. ## indicates a significant difference from the control (*P* < 0.01). ∗∗ indicates a significant difference from the 6-OHDA treatment alone (*P* < 0.01).

6-OHDA kills dopaminergic neurons by inducing oxidative stress through the increase of ROS production and the decrease of antioxidative enzyme activities in cells. We investigated the effect of NGR2 on 6-OHDA-induced oxidative stress in SH-SY5Y cells. The exposure of SH-SY5Y cells to 6-OHDA increased H2DCFDA fluorescence and MDA production compared with the control cells. This finding indicated that 6-OHDA elevated ROS production and lipid peroxidation in SH-SY5Y cells. 6-OHDA treatment also decreased levels of nuclear Nrf2 accumulation and the activities of HO-1, GSH-PX, and GR in SH-SY5Y cells. However, these effects were significantly suppressed by NGR2 pre-incubation (Figure 5(a)). These results suggested that the enhanced cellular levels of phase II detoxifying enzymes by NGR2 provided neuroprotection against 6-OHDA-induced oxidative stress.

### 3.6. NGR2 Reversed 6-OHDA-Induced Depolarization of Mitochondrial Membrane in SH-SY5Y Cells

Mitochondrial membrane depolarization is the hallmark of apoptosis. Thus, we used JC-1 to detect the mitochondrial membrane potential. In nonapoptotic cells, JC-1 emits green fluorescence as the monomeric form in cytosol and red fluorescence as aggregates in the mitochondria. In apoptotic cells, JC-1 emits only green fluorescence in cytosol. Therefore, mitochondrial membrane depolarization can be detected as a reduction in the ratio of the red-to-green fluorescence intensity. In this study, the treatment of SH-SY5Y cells with 6-OHDA resulted in a significant decrease in the ratio of red-to-green fluorescence intensity. This decrease suggested that 6-OHDA could depolarize the mitochondrial membrane of SH-SY5Y cells. However, NGR2 pre-incubation prevented this 6-OHDA effect. NGR2 treatment alone did not change the red-to-green fluorescence intensity ratio (Figures 5(b) and 5(c)). These results demonstrated that NGR2 could reverse the 6-OHDA-induced depolarization of the mitochondrial membrane in SH-SY5Y cells.

### 3.7. NGR2 Inhibited 6-OHDA-Induced Deregulation of Apoptosis-Related Proteins in SH-SY5Y Cells

We examined the markers of the apoptotic pathway to delineate the mechanism for NGR2-mediated neuroprotection.  Figure 5(d) shows that 6-OHDA treatment significantly increased the release of cytochrome c and the expression of cleaved caspase-9 and cleaved caspase-3. The expression of p-BAD in SH-SY5Y cells decreased compared with that of the control cells. However, NGR2 pre-incubation markedly blocked these effects. This finding suggested that NGR2-mediated neuroprotection was associated with the inhibition of 6-OHDA-induced altered expression of apoptosis-related proteins.

### 3.8. Dependence and Independence of NGR2-Mediated P90RSK and Nrf2 Activation on MEK1/2-ERK1/2 Pathways and JNK, P38, or PI3K/Akt Pathways

**Figure 6:**
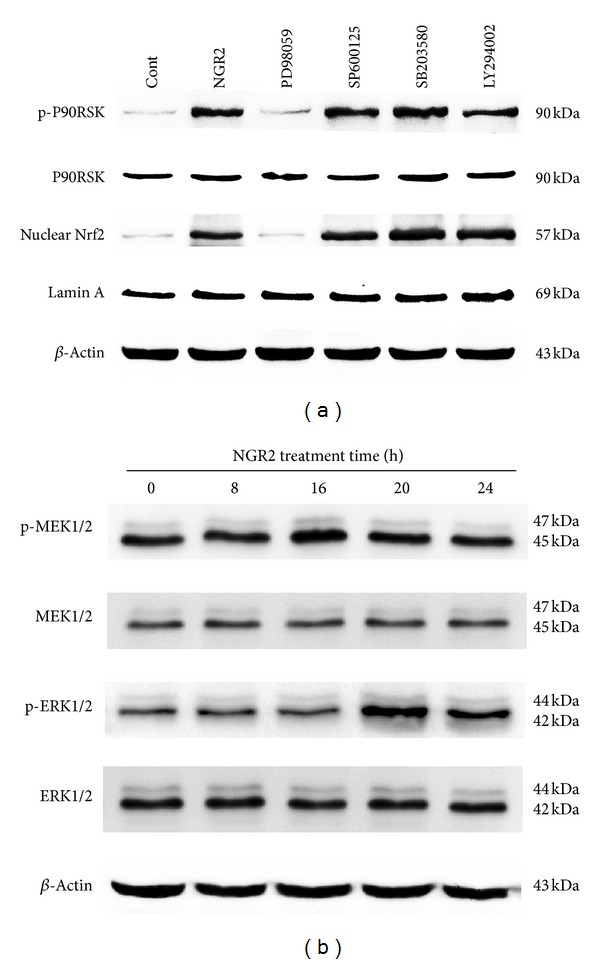
NGR2-mediated activation of P90RSK and Nrf2 was dependent of MEK1/2-ERK1/2 pathways but independent on JNK, P38, or PI3K/Akt pathways. The expression of proteins was determined by western blot analysis. The SH-SY5Y cells were preincubated with different inhibitors for 1 h followed by treatment with 20 *μ*M NGR2 for 24 h. (a) NGR2-mediated the phosphorylation of P90RSK and the nuclear Nrf2 accumulation were effectively prevented by MEK inhibitor PD98059 but not by JNK inhibitor SP600125, p38 inhibitor SB203580, or PI3K/Akt inhibitor LY294002. (b) Treatment of SH-SY5Y cells with 20 *μ*M NGR2 for different periods of time (4, 8, 16, 24 h) resulted in the activation of MEK1/2-ERK1/2 pathways.

To delineate the pathway involved in NGR2-mediated activation of P90RSK and Nrf2, we pretreated the SH-SY5Y cells with various inhibitors for 2 h, followed by incubation of NGR2 for 24 h. The MEK1/2 inhibitor PD98059 (20 *μ*M) effectively prevented the phosphorylation of P90RSK and nuclear Nrf2 accumulation mediated by NGR2 (Figure 6(a)). However, the JNK inhibitor SP600125 (20 *μ*M), p38 inhibitor SB203580 (20 *μ*M), and PI3K/Akt inhibitor LY294002 (10 *μ*M) all failed to affect the NGR2-mediated phosphorylation of P90RSK and nuclear Nrf2 accumulation. We then investigated the time course of MEK1/2 and ERK1/2 phosphorylation after NGR2 incubation. The results depicted in Figure 6(b) show that the treatment of SH-SY5Y cells with 20 *μ*M NGR2 for 24 h resulted in the phosphorylation of MEK1/2 and ERK1/2. MEK1/2 phosphorylation reached a peak point at 16 h. This finding was similar to ERK1/2 phosphorylation, which reached maximal elevation at 20 h. The aforementioned results indicated the involvement of MEK1/2-ERK1/2 but not of JNK, p38, or PI3K/Akt in NGR2-mediated P90RSK and Nrf2 activation.

### 3.9. NGR2-Mediated Neuroprotective Effects by P90RSK and Nrf2 Activation via MEK1/2-ERK1/2 Pathways

**Figure 7:**
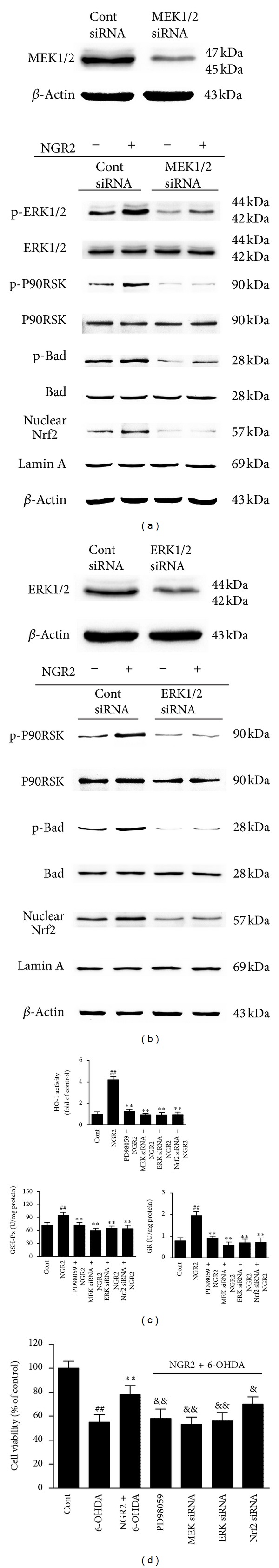
Neuroprotection of NGR2 involved the activation of P90RSK and Nrf2 through MEK1/2-ERK1/2 pathway. Protein expression was determined by western blot analysis. Both MEK1/2 siRNA (a) and ERK1/2 siRNA (b) suppressed NGR2-mediated activation of P90RSK and Nrf2. (c) NGR2-mediated activity enhancement of HO-1, GSH-PX, and GR was abolished by transfection of SH-SY5Y cells with MEK1/2 siRNA, ERK1/2 siRNA, and Nrf2 siRNA and by MEK inhibitor PD98059. The results are expressed as the mean ± SD of three independent experiments. ## indicates a significant difference from the control (*P* < 0.01). ∗∗ indicates a significant difference from the NGR2 treatment alone (*P* < 0.01). (d) The protective effect of NGR2 against 6-OHDA was partly abolished by Nrf2 siRNA and completely abolished by MEK1/2 siRNA, ERK1/2 siRNA, and PD98059. The results are expressed as the mean ± SD of three independent experiments. ## indicates a significant difference from the control (*P* < 0.01). ∗∗ indicates a significant difference from the 6-OHDA treatment alone (*P* < 0.01). & indicates a significant difference from NGR2+6-OHDA group (*P* < 0.05). && indicates a significant difference from NGR2+6-OHDA group (*P* < 0.01).

MEK1/2 siRNA and ERK1/2 siRNA were used to determine whether P90RSK and Nrf2 activation occurs downstream of the MEK1/2-ERK1/2 pathway. When SH-SY5Y cells were transfected with MEK1/2 siRNA, MEK1/2 expression in SH-SY5Y cells was successfully blocked (Figure 7(a)). Moreover, NGR2-mediated ERK1/2 phosphorylation, P90RSK phosphorylation, and BAD phosphorylation, as well as Nrf2 activation and the activity enhancement of phase II detoxifying enzymes (i.e., HO-1, GSH-PX, and GR), were all simultaneously inhibited (Figures 7(a) and 7(c)). Similarly, increased ERK1/2, p-P90RSK, and p-BAD expressions, as well as nuclear Nrf2 accumulation and the NGR2-mediated activity enhancement of HO-1, GSH-PX, and GR, were all effectively diminished by the suppression of ERK1/2 expression with siRNA (Figures 7(b) and 7(c)). The NGR2-mediated activity enhancement of HO-1, GSH-PX, and GR was also abolished by the transfection of SH-SY5Y cells with Nrf2 siRNA (Figure 7(c)). These results were corroborated by cell counting kit-8 test, where the protective effect of NGR2 against 6-OHDA was completely abolished by the genetic silencing of MEK1/2 and ERK1/2 and the pharmacologic blockade of MEK1/2 by using PD98059. However, the protective effect of NGR2 was partly inhibited by Nrf2 siRNA (Figure 7(d)).

## 4. Discussion

PD is a neurodegenerative disease that causes the selective loss of dopaminergic neurons in the substantia nigra. Although the etiology and pathogenesis of PD are not completely elucidated, accumulating evidence indicates that 6-OHDA, a hydroxylated dopamine metabolite, contributes to neuronal cell death in PD [22, 23].

6-OHDA can selectively kill dopaminergic neurons because of its high affinity to the dopamine transporter [24]. Once inside the neuron, 6-OHDA undergoes auto-oxidation or metabolic degradation and produces hydrogen peroxide, superoxide, and hydroxyl radicals. This process causes lipid peroxidation, protein oxidation, and DNA oxidation and finally results in oxidative stress, mitochondrial dysfunction, and apoptosis [3, 25]. 6-OHDA is widely accepted as a toxin for induction of the PD model *in vivo* and *in vitro* [23, 26, 27]. We investigated the neuroprotective effect of NGR2 and underlying mechanisms by using an *in vitro* model of 6-OHDA-induced cell death in SH-SY5Y cells.

6-OHDA caused oxidative stress in SH-SY5Y cells, which is consistent with the results of a previous study [28]. Oxidative stress occurs when ROS production overwhelms the antioxidative ability in cells. In this study, a significant increase in ROS production and MDA levels and a striking decrease in the activities of phase II detoxifying enzymes (HO-1, GSH-PX, and GR) were observed in 6-OHDA-treated cells. However, these 6-OHDA effects were suppressed by NGR2 preincubation.

Phase II detoxifying enzymes have a central function in neuronal defense against oxidative stress. The induction of phase II detoxifying enzymes also contributes to neuroprotection. HO-1 is a rate-limiting enzyme that catalyzes the oxidative catabolism of heme and produces biliverdin, carbon monoxide, and ferrous iron. GSH-PX can catalyze the reaction between reduced glutathione and hydrogen peroxide and convert reduced glutathione to oxidized glutathione. GR can convert oxidized glutathione to reduced glutathione. A cycle exists between reduced glutathione and oxidized glutathione by GSH-PX and GR. These phase II detoxifying enzymes generate potent antioxidative abilities. The expression of HO-1, GSH-PX, and GR is regulated by the activation of an important transcription factor Nrf2. In normal status, Nrf2 is sequestrated in the cytoplasm with Keap1 (Nrf2-Kelch-like ECH-associated protein 1). When activated, Nrf2 detaches itself from the Nrf2-Keap1 complex and translocates to the nucleus. Then, Nrf2 binds to the antioxidant responsive element and initiates the expression of phase II detoxifying enzymes [29]. We found that the nuclear translocation of Nrf2 in SH-SY5Y cells time dependently increased in response to NGR2 stimulation. The abilities of HO-1, GSH-PX, and GR all increased in a time-dependent manner by NGR2 incubation. To determine whether the elevation of HO-1, GSH-PX, and GR activities is dependent on Nrf2 activation, we transfected SH-SY5Y cells with Nrf2 siRNA. The NGR2-mediated upregulation of HO-1, GSH-PX, and GR activities was effectively inhibited by Nrf2 siRNA. The neuroprotective effect of NGR2 was also partly suppressed by Nrf2 siRNA. Moreover, nuclear Nrf2 accumulation and the activities of HO-1, GSH-PX, and GR were all decreased by 6-OHDA. However, NGR2 preincubation effectively inhibits the effects of 6-OHDA. These results suggested that the neuroprotective effect of NGR2 pre-incubation may be partly caused by Nrf2-dependent activity enhancement of HO-1, GSH-PX, and GR.

The results agreed well with a previous study in which 6-OHDA resulted in mitochondrial dysfunction and apoptosis in SH-SY5Y cells [25]. This phenomenon was manifested by mitochondrial membrane depolarization, cytochrome c release, caspase-9 and caspase-3 activation, DNA fragmentation, and increased apoptosis rate. However, NGR2 pre-incubation was effective in protecting SH-SY5Y cells against 6-OHDA-induced apoptosis. This is the first study to report the neuroprotective effect of NGR2 against 6-OHDA-induced apoptosis. The deregulation of Bcl-2 family protein is responsible for mitochondrial dysfunction induced by 6-OHDA. In the presence of apoptotic factors, the proapoptotic protein BAD interacts with the antiapoptotic protein Bcl-xl, thereby releasing the proapoptotic protein BAX from the Bcl-xl-BAX complex. BAX can disrupt the mitochondrial membrane, thus leading to the release of cytochrome c from the mitochondria. Consequently, caspase-9 and caspase-3 are subsequently activated, and apoptosis is initiated [30]. However, BAD can be inhibited when it is phosphorylated at serine 112. Therefore, promoting the phosphorylation of BAD at serine 112 contributed to apoptosis inhibition. A previous study showed that P90RSK could phosphorylate and inhibit BAD [31]. Therefore, the effect of NGR2 on P90RSK and BAD was investigated. NGR2 time dependently activated P90RSK and increased BAD phosphorylation, thereby preventing 6-OHDA-induced apoptotic cell death. To our knowledge, this is the first study that involves P90RSK in the neuroprotection against 6-OHDA-induced apoptosis.

Given that NGR2 activated Nrf2 and P90RSK in a time-dependent manner, the fact that NGR2-mediated protection was achieved by pre-incubation only rather than cotreatment with 6-OHDA was not surprising. This result may be attributed to the requirement of sufficient time for transcriptional and translational alterations to activate Nrf2 and P90RSK.

However, the mechanism of the activation of Nrf2 and P90RSK is still unclear. Previous reports showed that ERK1/2, JNK, P38, and PI3K/Akt pathways might be involved in P90RSK or Nrf2 activation in various cell types [14–20]. To ascertain which pathway participated in the activation of P90RSK and Nrf2, different inhibitors including MEK1/2 inhibitor PD98059, JNK inhibitor SP600125, p38 inhibitor SB203580, and PI3K/Akt inhibitor LY294002 were used in the present study. We found that the MEK1/2 inhibitor PD98059, rather than inhibitors against JNK, p38, or PI3 K/Akt, achieved a nearly complete inhibition of P90RSK and Nrf2 activation. This finding suggested that MEK1/2 was involved in NGR2-mediated P90RSK and Nrf2 activation.

MEK1/2, which is located upstream of ERK1/2, plays an important role in a variety of biological responses such as cell differentiation, proliferation, survival, and apoptosis [32]. Once activated, ERK1/2 leads to P90RSK phosphorylation and Nrf2 activation. We then explored the duration of MEK1/2 and ERK1/2 phosphorylations after NGR2 incubation. The treatment of SH-SY5Y cells with NGR2 led to MEK1/2 and ERK1/2 activation. The phosphorylation of MEK1/2 and ERK1/2 reached their respective peak points chronologically at 16 and 20 h. The question remains on whether ERK1/2 was activated by MEK1/2 and whether P90RSK and Nrf2 activation occurs downstream of the ERK1/2 pathway. To clarify this issue, SH-SY5Y cells were transfected with MEK1/2 siRNA or ERK1/2 siRNA. MEK1/2 siRNA simultaneously blocked NGR2-mediated the activation of ERK1/2, P90RSK, and Nrf2 as well as the subsequent activation of their respective downstream targets. NGR2-mediated activation of P90RSK and Nrf2, and the subsequent activation of their respective downstream targets were also simultaneously reversed by ERK1/2 siRNA. Moreover, NGR2-mediated neuroprotection was partly inhibited by Nrf2 siRNA and completely abolished by the genetic silencing of MEK1/2 and ERK1/2 or the application of a pharmacological inhibitor against MEK1/2. These results suggested that the immediate upstream activator of P90RSK and Nrf2 was ERK1/2, and the immediate upstream activator of ERK1/2 was MEK1/2, and these factors were all involved in NGR2-mediated neuroprotection either directly or indirectly.

Although P90RSK and Nrf2 activation was clearly activated by MEK1/2-ERK1/2 signaling pathways, the occurrence of a cross-talk between P90RSK and Nrf2 was not clearly elucidated. Detailed experiments are needed to investigate the exact underlying mechanism.

However, the successful application of the protective effect of NGR2 on different neuronal cell types remains to be determined. In this study, the neuroprotective effects of NGR2 against 6-OHDA toxic effects were confirmed in PC12 cells and rat primary cortical neurons. These results suggested that the neuroprotection of NGR2 was independent of cell type.

## 5. Conclusion 

**Figure 8:**
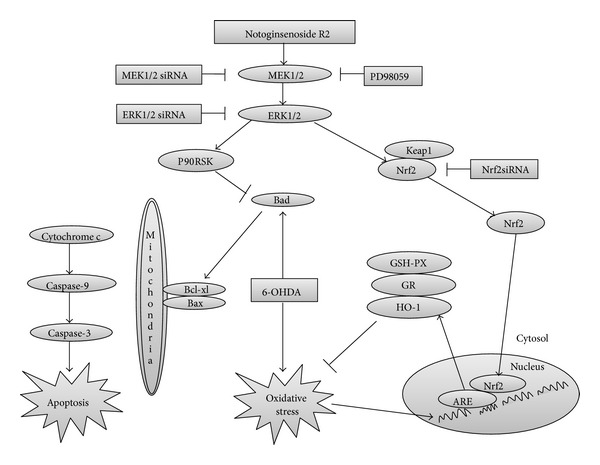
Schematic of the neuroprotective effects of NGR2 against 6-OHDA-induced neurotoxicity. NGR2 activates P90RSK and Nrf2 via MEK1/2-ERK1/2 pathways. P90RSK activation could inhibit BAD, thereby inhibiting 6-OHDA-induced mitochondrial membrane depolarization, cytochrome c release, caspase-9 and caspase-3 activation, and apoptosis. Nrf2 activation could enhance the activities of phase II detoxifying enzymes such as HO-1, GSH-PX, and GR. This phenomenon suppressed 6-OHDA-induced oxidative stress and DNA fragmentation.

NGR2 exhibited neuroprotective effects against 6-OHDA-induced apoptosis in SH-SY5Y cells. The mechanism of this neuroprotection was associated with NGR2-mediated P90RSK and Nrf2 activation via MEK1/2-ERK1/2 pathways (Figure 8). NGR2 provides a potential promising alternative option as adjunctive medication for the treatment of PD in clinic.
