# Correction to “P90RSK and Nrf2 Activation via MEK1/2‐ERK1/2 Pathways Mediated by Notoginsenoside R2 to Prevent 6‐Hydroxydopamine‐Induced Apoptotic Death in SH‐SY5Y Cells”

**DOI:** 10.1155/ecam/9810324

**Published:** 2026-02-11

X.‐B. Meng, G.‐B. Sun, M. Wang, J. Sun, M. Qin, and X.‐B. Sun, “P90RSK and Nrf2 Activation via MEK1/2‐ERK1/2 Pathways Mediated by Notoginsenoside R2 to Prevent 6‐Hydroxydopamine‐Induced Apoptotic Death in SH‐SY5Y Cells,” *Evidence-Based Complementary and Alternative Medicine*, 2013, 971712, https://doi.org/10.1155/2013/971712.

In the article, there is an error in Figure 3a. The incorrect panel for sole NGR2 treatment was selected during manuscript preparation, and the correct Figure 3 is shown below.

Figure 3Protective effect of NGR2 on 6‐OHDA‐induced apoptosis in SH‐SY5Y cells. The SH‐SY5Y cells were preincubated with 20 μM NGR2 for 24 h followed by treatment with 50 μM 6‐OHDA for 24 h. (a) Photographs of morphological changes in SH‐SY5Y cells were visualized by an inverted microscope connected to a digital camera, bar = 50 μm. (b) Photographs of DNA fragmentation were detected by TUNEL assay in the apoptotic SH‐SY5Y cells, bar = 50 μm. Arrows represent TUNEL‐positive cells. (c) Cell apoptosis was determined by Annexin V‐PI double staining kits by using flow cytometry. (d) Quantification of the TUNEL‐positive cell rate. (e) Quantification of the apoptosis rate. The results were expressed as the mean ± SD of three independent experiments. ## indicates a significant difference from the control (*p* < 0.01). ∗∗ indicates a significant difference from the 6‐OHDA treatment alone (*p* < 0.01).

**Figure:**
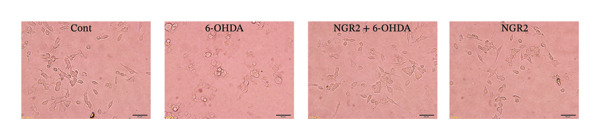
(a)

**Figure:**
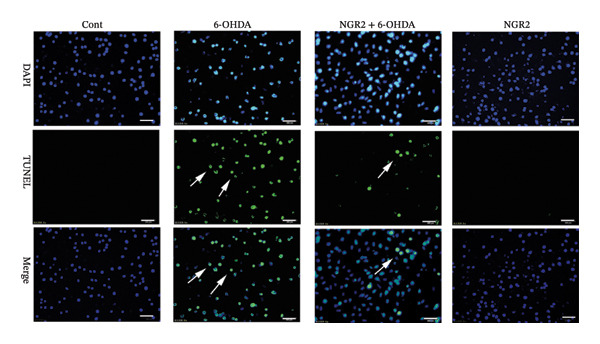
(b)

**Figure:**
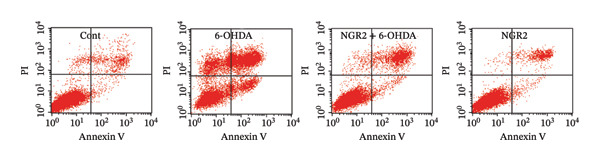
(c)

**Figure:**
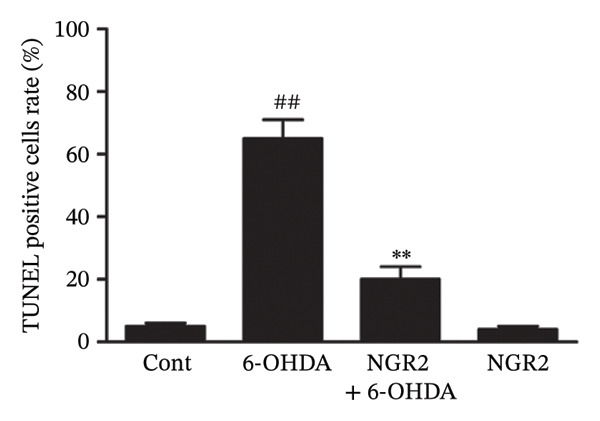
(d)

**Figure:**
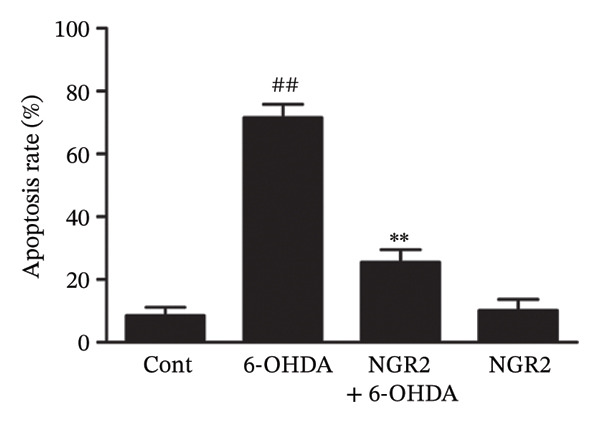
(e)

We apologize for this error.

